# Embryonic origins of forebrain oligodendrocytes revisited by combinatorial genetic fate mapping

**DOI:** 10.7554/eLife.95406

**Published:** 2024-09-11

**Authors:** Yuqi Cai, Zhirong Zhao, Mingyue Shi, Mingfang Zheng, Ling Gong, Miao He

**Affiliations:** 1 Institutes of Brain Science, State Key Laboratory of Medical Neurobiology and MOE Frontiers Center for Brain Science, Department of Neurobiology, Zhongshan Hospital, Fudan University Shanghai China; University of California, San Francisco United States; University of Barcelona Spain

**Keywords:** oligodendrocyte, developmental origin, genetic fate mapping, Mouse

## Abstract

Multiple embryonic origins give rise to forebrain oligodendrocytes (OLs), yet controversies and uncertainty exist regarding their differential contributions. We established intersectional and subtractional strategies to genetically fate map OLs produced by medial ganglionic eminence/preoptic area (MGE/POA), lateral/caudal ganglionic eminences (LGE/CGE), and dorsal pallium in the mouse brain. We found that, contrary to the canonical view, LGE/CGE-derived OLs make minimum contributions to the neocortex and corpus callosum, but dominate piriform cortex and anterior commissure. Additionally, MGE/POA-derived OLs, instead of being entirely eliminated, make small but sustained contribution to cortex with a distribution pattern distinctive from those derived from the dorsal origin. Our study provides a revised and more comprehensive view of cortical and white matter OL origins, and established valuable new tools and strategies for future OL studies.

## Introduction

Oligodendrocytes (OLs) are an important class of macroglia responsible for producing the myelin sheaths that insulate and protect neuronal axons. Forebrain OLs arise from multiple embryonic origins. Previous fate-mapping study using *Nkx2.1^Cre^* (Xu et al., 2008), *Gsh2^Cre^* (Kessaris et al., 2006), and *Emx1^Cre^* (Gorski et al., 2002) reported consecutive and competing waves of OLs derived from medial ganglionic eminence/preoptic area (MGE/POA), lateral/caudal ganglionic eminences (LGE/CGE), and dorsal pallium (Kessaris et al., 2006). The first wave of OLs generated by MGE/POA (_MP_OLs) was believed to be eliminated postnatally, while those from the second and third waves (_LC_OLs and dOLs) survive and populate the cortex and corpus callosum at comparable proportions. Several other studies provided both supporting and contradicting evidence to this model (Nakahira et al., 2006; Tsoa et al., 2014; Naruse et al., 2016; Orduz et al., 2019; Liu et al., 2021; Shen et al., 2021; Tripathi et al., 2011; Winkler et al., 2018). Moreover, *Gsh2* was recently found to be expressed in dorsal progenitors (Zhang et al., 2020), casting doubt on the interpretation of lineage tracing data from *Gsh2^Cre^*.

In this study, we generated new genetic tools and combinatorial fate-mapping strategies which allow direct visualization and comparison among OLs derived from different origins. We found that neocortical OLs are primarily composed of dOLs, rather than similar proportions of _LC_OLs and dOLs. In contrast, _LC_OLs and dOLs made comparable contributions to piriform cortex. We also found that although _MP_OLs only make a small contribution, they do persist in the cortex beyond adulthood with a unique spatial pattern distinct from that of the dOLs. In the two major white matter commissure tracts, dOLs are the vast majority in corpus callosum but make little contribution to anterior commissure, while _LC_OLs behaved the opposite. These findings significantly revised the classical view and provided a new and more comprehensive picture of cortical and white matter OL origins.

## Results and discussion

**Figure 1.**
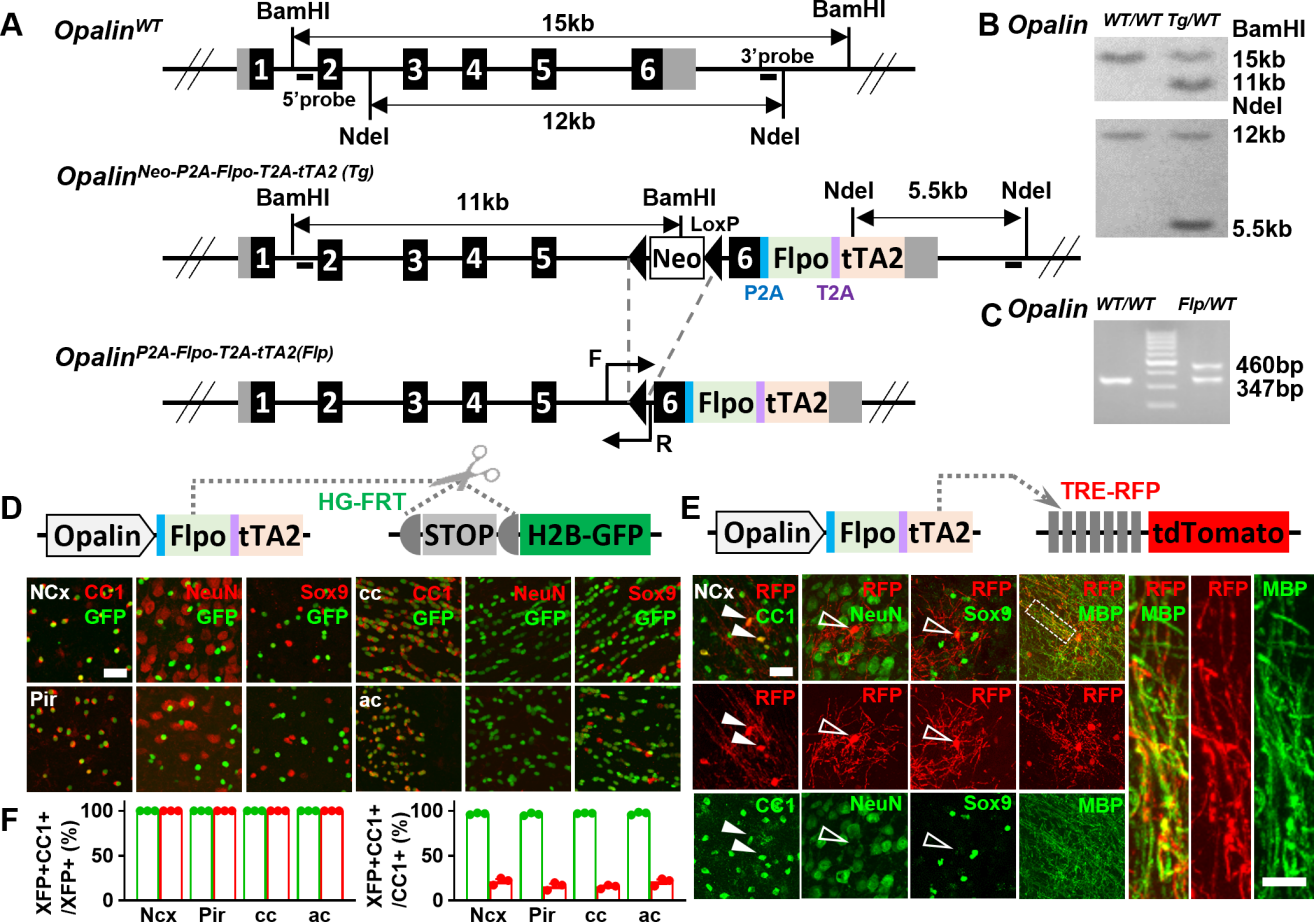
A new driver mouse for efficient and specific oligodendrocyte (OL) labeling. (**A**) Scheme for generating the *Opalin^P2A-Flpo-T2A-tTA2^* allele. (**B**) Southern blot confirmation of correctly targeted embryonic stem cell clone. (**C**) Genomic polymerase chain reaction (PCR) to genotype F1 offspring. (**D**) OL labeling by Flp. (**E**) OL labeling by tTA2. High magnification images of the boxed region showing co-localization of red fluorescent protein (RFP) with myelin basic protein (MBP) staining, which further demonstrated the myelination ability of labeled OLs. (**F**) Quantification of labeling specificity (left panel) and efficiency (right panel) by colacalization with OL marker CC1. Both reporting systems are highly specific, as shown by the complete co-localization of fluorescent protein (XFP) with OL marker (CC1) and lack of co-staining with neuronal marker (NeuN) or astrocyte marker (Sox9). Quantification bar graph was not presented for NeuN and Sox9 as zero co-localizations were observed in all analyzed regions. Close to complete OL labeling was achieved by Flp-dependent H2B-GFP reporter in all analyzed regions (green dots), while sparser labeling with variable regional density was achieved by tTA2-dependent tdTomato reporter driven by TRE promoter (red dots). NCx: neocortex. Pir: piriform cortex. cc: corpus callosum. ac: anterior commissure. Scale bar: 50 μm in low magnification images, 5 μm in high magnification images. Quantification: *n* = 3. Dots represent data from individual mice. Figure 1—source data 1.Raw unedited blot for Figure 1B. Figure 1—source data 2.Uncropped and labeled blot for Figure 1B. Figure 1—source data 3.Raw unedited gel for Figure 1C. Figure 1—source data 4.Uncropped and labeled gel for Figure 1C. Figure 1—source data 5.The raw data for the visualization of data presented in Figure 1F.

To unambiguously track OLs from different embryonic origins, we first generated a knock-in driver, *Opalin^P2A-Flpo-T2A-tTA2^* (Figure 1), orthogonal to Cre drivers that label dorsal or ventral progenitors (*Progenitor^Cre^*). *Opalin* (also known as *Tmem10*) encodes oligodendrocytic myelin paranodal and inner loop protein that are specifically expressed in differentiated OLs (Kippert et al., 2008; Yoshikawa et al., 2008; Jiang et al., 2013; Marques et al., 2016). In *Opalin^P2A-Flpo-T2A-tTA2^*, Flpo and tTA2 were inserted before the STOP codon and linked by self-cleavage peptide P2A and T2A (Figure 1A–C), allowing co-transcription and translation with *Opalin*. Flp-mediated recombination by this driver (hereinafter referred to as *Opalin^Flp^* for simplicity) enables highly specific, efficient, and irreversible OL labeling, while the tTA2 component offers the flexibility for OL-specific labeling in tunable densities (Figure 1D–F).

**Figure 2.**
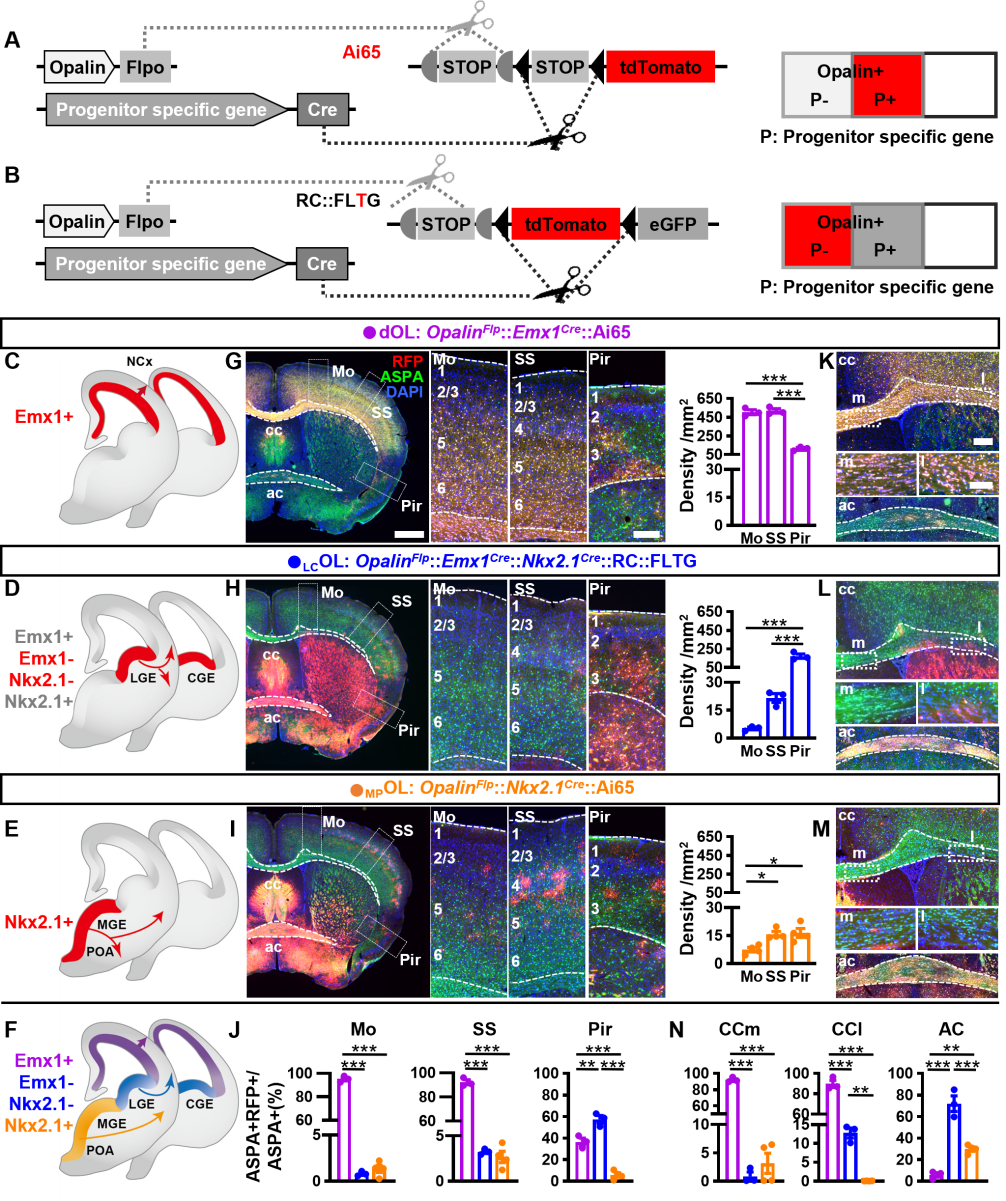
Combinatorial fate mapping of dOLs, _MP_OLs, and _LC_OLs. (**A**) Strategy for intersectional labeling. Flp-AND-Cre labels oligodendrocytes (OLs) from Cre-expressing progenitors with RFP. (**B**) Strategy for subtractional labeling of OLs derived from non-Cre-expressing progenitors with RFP. The eGFP expressing OLs derived from Cre-expressing progenitors were not used for analysis in this scenario and thereby were not highlighted by color. Schematics showing intersectional labeling of dOLs in *Opalin^Flp^::Emx1^Cre^*::Ai65 (**C**), subtractional labeling of _LC_OLs in *Opalin^Flp^::Emx1^Cre^::Nkx2.1^Cre^*::RC::FLTG (**D**), intersectional labeling of _MP_OLs in *Opalin^Flp^::Nkx2.1^Cre^*::Ai65 (**E**), and cortical OLs derived from all three origins (**F**). (**G–I**) Representative images (left panels) and quantifications (right panels) of RFP+ cell density in motor cortex (Mo), somatosensory cortex (SS), and piriform cortex (Pir). (**J**) Quantification of differential contribution to ASPA+ OLs by three embryonic origins to Mo, SS, and Pir. Representative images (**K–M**) and quantifications (**N**) of differential contribution to ASPA+ OLs by three embryonic origins in the two major commissure white matter tracts: corpus callosum (cc) and anterior commissure (ac). _MP_OLs and _LC_OLs preferentially reside in the medial and lateral cc (cc-m and cc-l), respectively. Scale bar: 1 mm in low magnification images in (**G–I**), 250 μm in high magnification images of the boxed area in (**G–I**) and low magnification images in (**K–M**), 100 μm in high magnification images of the boxed area (cc-m and cc-l) in (**K–M**). *n* = 3 for dOLs and _LC_OLs; *n* = 4 for _MP_OLs. Dots represent data from individual mice. Error bar: standard error of the mean (SEM). *p < 0.05, **p < 0.01, ***p < 0.001. Figure 2—source data 1.The raw data for the visualization of data presented in Figure 2G–J, N.

Next, we established two types of genetic combinatorial fate-mapping strategies to directly visualize OLs from different embryonic origins (Figure 2): (1) combining *Opalin^Flp^* and *Progenitor^Cre^* with intersectional reporters Ai65 to label OLs derived from Cre+ progenitor domain by RFP (Figure 2A); (2) combining *Opalin^Flp^* and *Progenitor^Cre^* with RC::FLTG (Plummer et al., 2015) to simultaneously label OLs derived from Cre+ progenitors by green fluorescent protein (GFP) and OLs derived from the complementing Cre− progenitors by RFP (Figure 2B, Figure 2—figure supplement 1A). The first approach allowed us to track dOLs and _MP_OLs (Figure 2C, E). The second approach empowered us to observe and compare OLs generated from dorsal and ventral origins (Figure 2—figure supplement 1B), or those from Gsh2+ and Gsh2− progenitors, in the same brain (Figure 2—figure supplement 1C). Importantly, the subtraction power enabled us to target OLs derived from LGE/CGE progenitors that express neither *Emx1* nor *Nkx2.1* (Figure 2D and Figure 2—figure supplement 1D). In addition, these strategies greatly facilitated the identification of OLs derived from specific origin which exist at relatively low density in certain regions.

**Figure 2—figure supplement 1.**
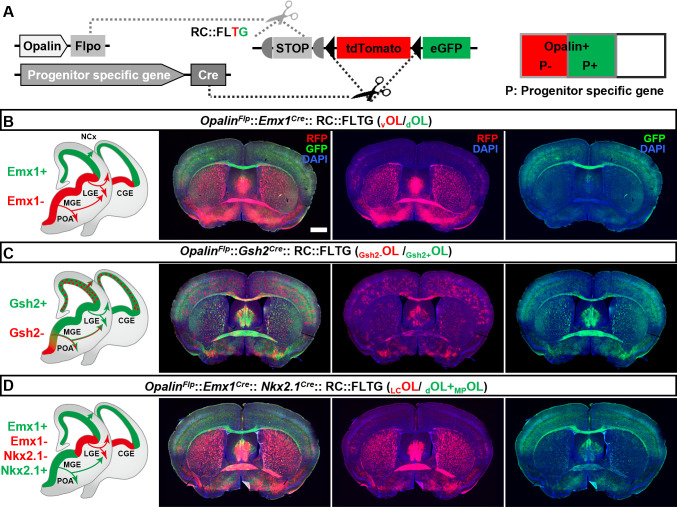
Simultaneous differential labeling of oligodendrocytes (OLs) derived from complementary embryonic origins. (**A**) Strategy for simultaneous labeling of OLs derived from complementary origins. Flp-NOT-Cre labels OLs from non-Cre-expressing progenitors with RFP, while Flp-AND-Cre labels OLs from Cre-expressing progenitors with eGFP. (**B**) Coronal sections showing GFP+ OLs from dorsal origin (dOLs) and RFP+ OLs from ventral origin (vOLs) in *Opalin^Flp^::Emx1^Cre^*::RC::FLTG. (**C**) Coronal sections showing GFP+ OLs derived from Gsh2+ progenitors (_Gsh2+_OL) and RFP+ OLs derived from Gsh2− progenitors (_Gsh2−_OL) in *Opalin^Flp^::Gsh2^Cre^*::RC::FLTG. (**D**) Coronal sections showing GFP+ OLs from dorsal and medial ganglionic eminence/preoptic area (MGE/POA) origin (dOLs+_MP_ OLs) and RFP+ OLs from lateral/caudal ganglionic eminences (LGE/CGE) origin (_LC_OLs) in *Opalin^Flp^*:: *Emx1^Cre^:: Nkx2.1^Cre^*::RC::FLTG. Scale bar: 1 mm.

**Figure 2—figure supplement 2.**
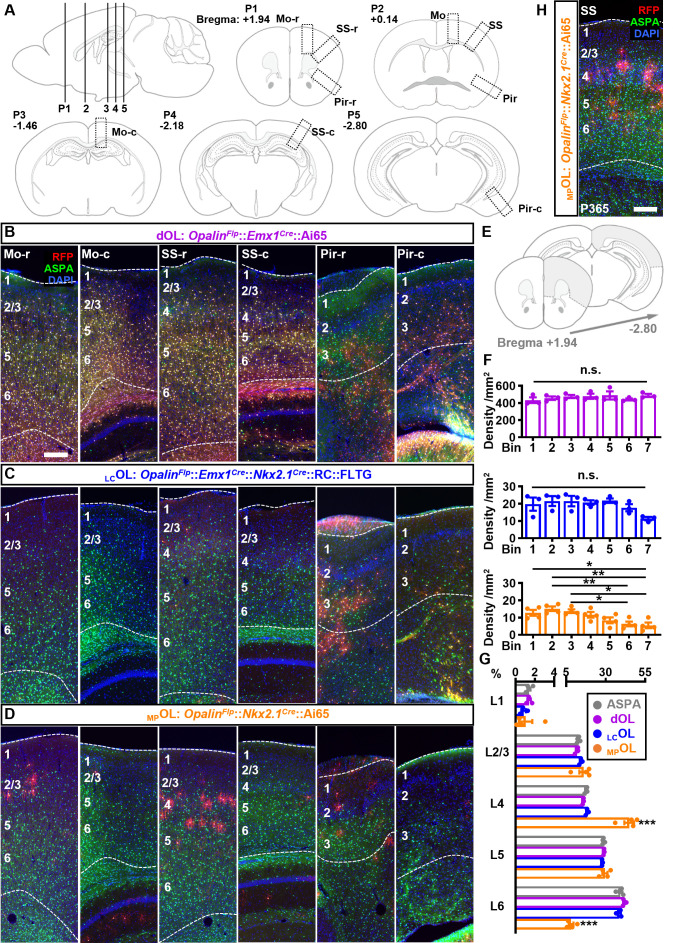
The distribution pattern of cortical dOLs, _MP_OLs, and _LC_OLs. (**A**) Schematics of cortical regions chosen for quantifications and for showing representative images (boxed regions). Every fourth coronal section between Bregma +1.94 and −2.80 mm was analyzed. Positions (P) 1–5 correspond to sections from which representative images were taken from. P2 corresponds to the sections shown in Figure 2G–I. (**B–D**) For each cortical region, two representative images at the rostral (r) and caudal (c) ends were presented for each combination. (**E, F**) Quantification of rostral–caudal distribution of neocortical dOLs, _LC_OLs, and _MP_OLs. Neocortical area of slices ranging from Bregma +1.94 to −2.80 (gray shaded region in **E**) was quantified and grouped into seven evenly divided bins along the rostral–caudal axis. Densities of dOLs and _LC_OLs showed no significant change across bins, while _MP_OLs exhibited lower density in more caudal regions. (**G**) Distributions of dOLs, _LC_OLs, and _MP_OLs across six layers in SS. Similar to the total oligodendrocyte (OL) distribution quantified based on aspartoacylase (ASPA) staining, more dOLs and _LC_OLs reside in deeper layers. In contrast, _MP_OLs are highly enriched in L4 at the cost of L6 with significant deviation from the total OLs. (**H**) Representative image of SS from 1-year-old *Opalin^Flp^::Nkx2.1^Cre^*::Ai65 mouse. Scale bar: 200 μm. *n* = 3 for dOLs and _LC_OLs; *n* = 4 for _MP_OLs and ASPA. Dots represent data from individual mice. Error bar: standard error of the mean (SEM). *p < 0.05, **p < 0.01, ***p < 0.001. Figure 2—figure supplement 2—source data 1.The raw data for the visualization of data presented in Figure 2—figure supplement 2F, G.

**Figure 2—figure supplement 3.**
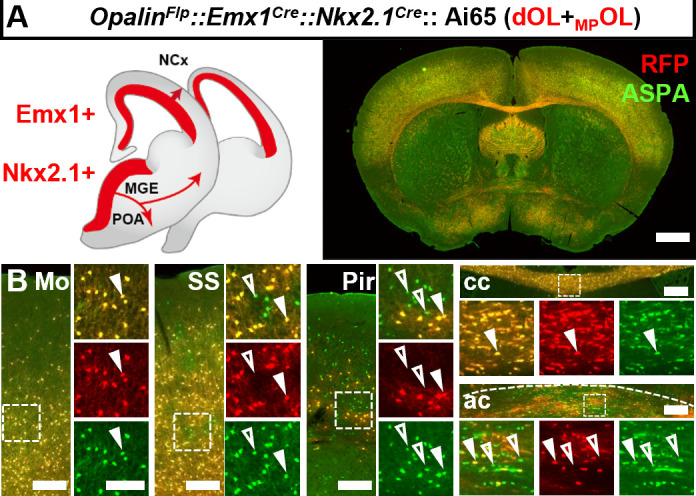
Intersectional labeling of oligodendrocytes (OLs) derived from both dorsal origin and medial ganglionic eminence/preoptic area (MGE/POA). (**A**) Coronal sections showing both dOLs and _MP_OLs labeled by RFP in *Opalin^Flp^::Emx1^Cre^::Nkx2.1^Cre^*::Ai65. (**B**) Higher magnification images showing ASPA+RFP+ dOLs/_MP_OLs (closed arrow heads) and ASPA+RFP− putative _LC_OLs (open arrow heads). The latter cells were difficult to find in neocortical regions such as motor cortex (Mo) and somatosensory cortex (SS), and corpus callosum (cc), but were frequently encountered in piriform cortex (Pir) and anterior commissure (ac). Scale bar: 1 mm in low magnification images, 200 μm in Mo, SS, cc, and ac, 10 μm in high magnification images of boxed area.

Deploying these strategies, we assessed the differential contributions of dOLs, _LC_OLs, and _MP_OLs by analyzing RFP+ cells in the following mice: *Opalin^Flp^::Emx1^Cre^*::Ai65 (Figure 2C), *Opalin^Flp^::Emx1^Cre^::Nkx2.1^Cre^*::RC::FLTG (Figure 2D), and *Opalin^Flp^::Nkx2.1^Cre^*::Ai65 (Figure 2E). To better assess their contributions to the total OL population (Figure 2F), we co-stained RFP with the mature OL marker aspartoacylase (ASPA) (Huang et al., 2023; Figure 2G–I) and quantified the ratio of co-localization (Figure 2J). Notably, all RFP+ cells are ASPA+, reassured the specificity of our label strategies. We observed two significant differences from the traditional model in the neocortex. The first major deviation is that, instead of comparable contributions by dOLs and _LC_OLs, the vast majority of neocortical OLs were dOLs but not _LC_OLs. The densities (Figure 2G–I and Figure 2—figure supplement 2A–F) and ASPA ratios (Figure 2J) of dOLs are much higher than those of _LC_OLs. Considering the possibility of incomplete recombination in combinatorial reporters, and the relatively low Cre activity in the dorsal MGE of *Nkx2.1^Cre^* (Xu et al., 2008), the genuine contribution of _LC_OLs to the neocortex could be even lesser than our current observation. Therefore, the large quantity of neocortical OLs labeled by *Gsh2^Cre^* in previous study (Kessaris et al., 2006) or by GFP in *Opalin^Flp^::Gsh2^Cre^*::RC::FLTG (Figure 2—figure supplement 1C) most likely were predominantly dOLs generated by Gsh2+ dorsal progenitors (Zhang et al., 2020), rather than bona fide _LC_OLs.

The second major deviation is that cortical _MP_OLs are not completely depleted postnatally. Instead, they make a small but continued contribution with a unique spatial distribution pattern (Figure 2I, J and Figure 2—figure supplement 2D–G). _MP_OLs display a clear rostrocaudal density decline (Figure 2—figure supplement 2E, F), a higher density in somatosensory cortex (SS) than motor cortex (Mo) (Figure 2I), and a laminar preference toward layer 4 (L4) in SS (Figure 2—figure supplement 2G). In contrast, the distribution of dOLs and _LC_OLs do not vary significantly across the rostrocaudal axis (Figure 2—figure supplement 2E, F) or between Mo and SS (Figure 2G, H), but exhibits increased density toward deeper layers (Figure 2—figure supplement 2G). Importantly, we have observed cortical _MP_OLs in mice as old as 1 year (Figure 2—figure supplement 2H), well beyond the age analyzed in previous reports (Kessaris et al., 2006; Orduz et al., 2019; Liu et al., 2021), suggesting a persisted contribution.

We then turned our attention to the lateral three-layer archicortex, piriform cortex (Pir). Different from the neocortex, Pir contains higher proportions (Figure 2J) of _LC_OLs than dOLs. _MP_OLs make the lowest contribution (Figure 2J) at a density similar to SS and higher than Mo (Figure 2I).

These combinatorial models also grant us the opportunity to revisit the differential contributions of dOLs, _LC_OLs, and _MP_OLs to the two commissural white matter tracts, corpus callosum (cc), and anterior commissure (ac), which contain high density of OLs (Figure 2K–M). We found that, similar to the neocortex, cc is mainly populated by dOLs and supplemented by very low proportions of _LC_OLs and _MP_OLs (Figure 2K–N). Interestingly, _LC_OLs and _MP_OLs seem to show preferential distribution in the lateral and medial regions of cc (cc-l and cc-m), respectively (Figure 2L–N). Different from cc, ac is mainly populated by _LC_OLs and _MP_OLs and supplemented by very low proportion of dOLs (Figure 2K–N).

To substantiate the above results, we further breed *Opalin^Flp^::Emx1^Cre^::Nkx2.1^Cre^*::Ai65 to label dOLs together with _MP_OLs by RFP and co-stained them with ASPA (Figure 2—figure supplement 3). RFP−ASPA+ cells were difficult to find in Mo, SS, and cc, but were more easily observed in Pir and ac, consistent with the respective low and high _LC_OL contributions in these regions.

**Figure 3.**
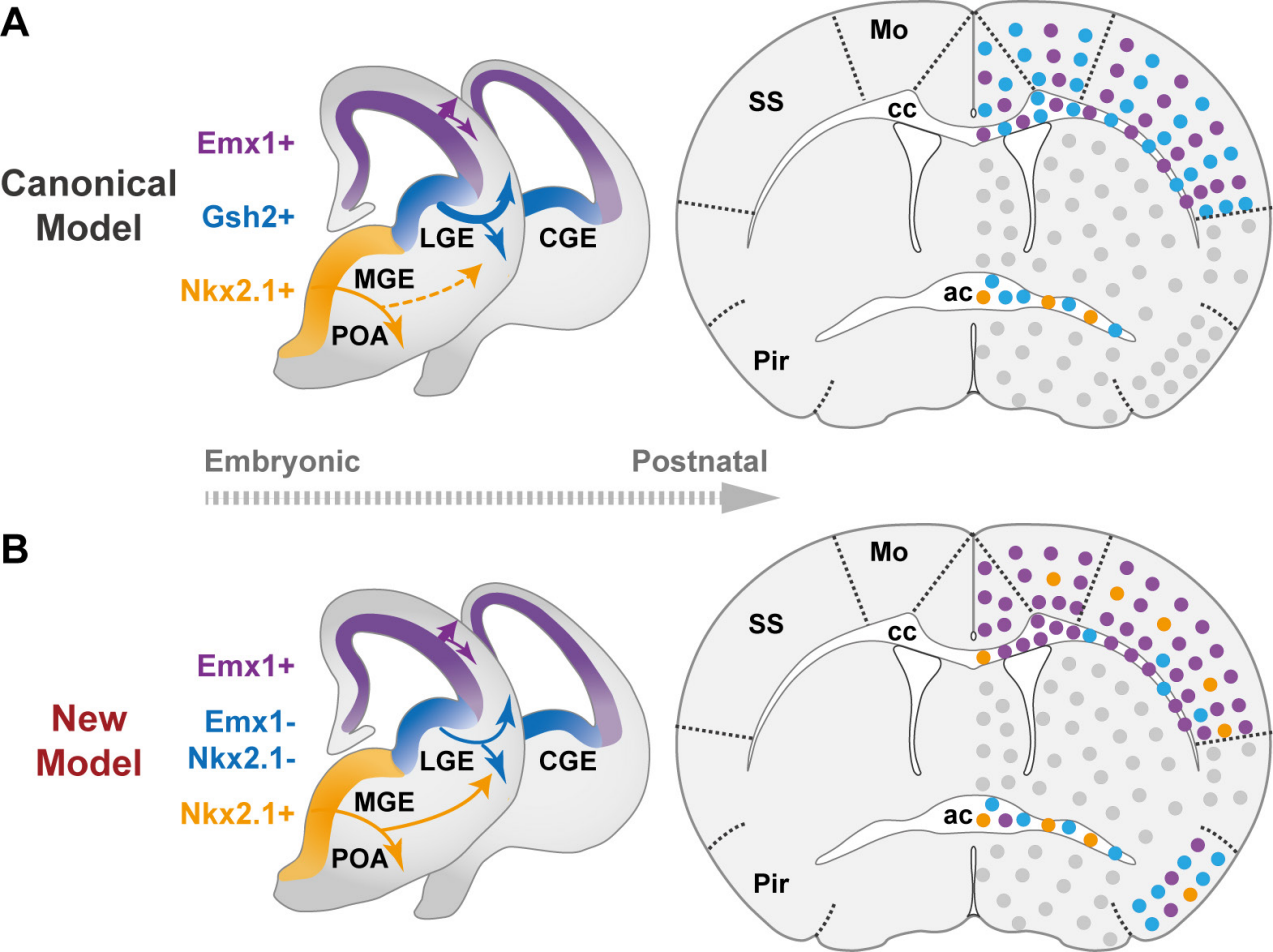
The classical and revised model of forebrain oligodendrocyte (OL) origins. (**A**) In the classical model (Kessaris et al., 2006), OLs derived from medial ganglionic eminence/preoptic area (MGE/POA) (orange) were largely eliminated postnatally (thin dashed line), while those from lateral/caudal ganglionic eminences (LGE/CGE) (blue) and dorsal origin (purple) survive at similar proportions (thick solid line). Therefore, neocortex (NCx) and corpus callosum (cc) contain comparable density of _LC_OLs (blue dots) and dOLs (purple dots) and are devoid of _MP_OLs (orange dots). (**B**) In the new model, NCx and cc mainly contain dOLs with very low contribution from the ventral origins. _LC_OLs mainly contribute to piriform cortex (Pir) and anterior commissure (ac). _MP_OLs makes a small but sustained contribution to NCx, with a strong laminar preference toward layer 4 in somatosensory cortex (SS). In addition, dOLs and _MP_OLs also make substantial contributions to Pir and ac, respectively. Gray dots indicate OLs in unanalyzed regions.

In summary, our findings significantly revised the canonical model of forebrain OL origins (Figure 3A), and provided a new and more comprehensive view (Figure 3B). We demonstrated that neocortical OLs are mainly derived from dorsal origin with small but lasting contribution from the ventral origin (Figure 2, Figure 2—figure supplements 1B and 2). Our data showed that LGE/CGE makes little contribution to neocortex and cc, but makes major contribution to piriform cortex and ac (Figure 2 and Figure 2—figure supplement 3). This finding is supported by another report in which in utero electroporation failed to label LGE-derived cortical OLs in both embryonic and early postnatal brains, and an exclusion strategy revealed very low percentage of LGE/CGE-derived cortical OLs in neonatal brains (Li et al., 2023). The lack of adult labeling in our study together with the lack of developmental labeling in the other study suggests that the lack of _LC_OL in neocortex is less likely caused by competitive postnatal elimination, but more likely due to limited production and/or allocation. We further discovered that MGE/POA makes a small but persistent contribution to the neocortex with a distinct distribution pattern featured by a rostral-high to caudal-low gradient and a preference toward L4 in SS (Figure 2—figure supplement 2). Whether their enduring existence and highly biased localization has functional implications awaits future exploration. In addition, we found that the cc showed a similar OL composition as the neocortex, but the Pir and the ac each exhibited distinct OL compositions in term of their embryonic origins. _LC_OLs are the major contributor to both regions, while dOLs and _MP_OLs mainly contribute to Pir and ac, respectively (Figure 2).

In addition to the new framework of forebrain OL origins (Figure 3), we also generated a new driver (Figure 1) and established multiple combinatorial genetic models (Figure 2) for efficient tracking and direct visualization of OLs from different embryonic origins without interference from other cells types sharing the same progenitor domains such as OL precursors, astrocytes, and neurons (Figures 1–2). These tools set up a firm foundation and will provide reliable experimental access for future inquiries on the development and function of diverse OLs in healthy and disease brains (Gong et al., 2022), especially to uncover the relationship between their developmental origins and the functional and molecular heterogeneity.

## Materials and methods

**Key resources table:** 

Reagent type (species) or resource	Designation	Source or reference	Identifiers	Additional information
Genetic reagent (*Mus musculus*)	*Nkx2.1^Cre^*	The Jackson Laboratory	Strain#: 008661; RRID: IMSR_JAX:008661	
Genetic reagent (*Mus musculus*)	*Gsh2^Cre^*	The Jackson Laboratory	Strain#: 025806; RRID: IMSR_JAX:025806	
Genetic reagent (*Mus musculus*)	*Emx1^Cre^*	The Jackson Laboratory	Strain#: 005628; RRID: IMSR_JAX:005628	
Genetic reagent (*Mus musculus*)	Ai65	The Jackson Laboratory	Strain#: 021875; RRID: IMSR_JAX:021875	
Genetic reagent (*Mus musculus*)	RC::FLTG	The Jackson Laboratory	Strain#: 026932; RRID: IMSR_JAX:026932	
Genetic reagent (*Mus musculus*)	Ai62	The Jackson Laboratory	Strain#: 022731; RRID: IMSR_JAX:022731	
Genetic reagent (*Mus musculus*)	HG-FRT	The Jackson Laboratory	Strain#: 028581; RRID: IMSR_JAX:028581	
Genetic reagent (*Mus musculus*)	*Opalin^P2A-Flpo-T2A-tTA2^*	This paper		See Materials and methods, Mice
Antibody	anti-RFP (goat polyclonal)	SICGEN	Cat# AB0081-200; RRID: AB_2333095	IF (1:2000)
Antibody	anti-RFP (rabbit polyclonal)	Rockland	Cat# 600-401-379; RRID: AB_2209751	IF (1:2000)
Antibody	anti-GFP (chicken polyclonal)	Aves Labs	Cat# GFP-1020; RRID: AB_10000240	IF (1:1000)
Antibody	anti-MBP (rat polyclonal)	AbD Serotec	Cat# MCA409S; RRID: AB_325004	IF (1:500)
Antibody	anti-CC1 (rabbit polyclonal)	Oasis Biofarm	Cat# OB-PRB070; RRID: AB_2934254	IF (1:500)
Antibody	anti-CC1 (mouse polyclonal)	Millipore	Cat# OP80; RRID: AB_2057371	IF (1:300)
Antibody	anti-ASPA (rat polyclonal)	Oasis Biofarm	Cat# OB-PRT005; RRID: AB_2938679	IF (1:200)
Antibody	anti-Sox9 (rabbit polyclonal)	Chemicon	Cat# AB5535; RRID: AB_2239761	IF (1:2000)
Antibody	anti-NeuN (mouse monoclonal)	Millipore	Cat# MAB377; RRID: AB_2298772	IF (1:500)
Sequence-based reagent	Opalin-F	This paper	PCR primers	GGCCTATGTTTGATTTCCAGCACTG
Sequence-based reagent	Opalin-R	This paper	PCR primers	AGCACTTATGACTGCTGAGCCGTTC
Chemical compound, drug	Tail lysis buffer	Viagen	Cat# 102-T	
Chemical compound, drug	Proteinase K	Beyotime	Cat# ST535	
Chemical compound, drug	Sodium pentobarbital	Sigma-Aldrich	Cat# P3761	
Chemical compound, drug	Normal Donkey Serum	Abcam	Cat# ab7475	
Chemical compound, drug	Triton X-100	Sigma-Aldrich	Cat# X100PC	
Chemical compound, drug	Citrate buffer	Oasis-Biofarm	Cat# BR-AB001	
Other	Aqua-mount	Southern Biotech	Cat# 0100-01	
Other	DAPI stain	Invitrogen	Cat# D1306	(10 mg/ml)
Software, algorithm	ImageJ	National Institutes of Health	RRID: SCR_003070	
Software, algorithm	QuPath	Queen’s University Belfast	RRID: SCR_018257	
Software, algorithm	Adobe Photoshop	Adobe Systems	RRID: SCR_014199	
Software, algorithm	GraphPad Prism v8.0.1	GraphPad Software	RRID: SCR_002798	

### Mice

All mouse studies were carried out in strict accordance with the guidelines of the Institutional Animal Care and Use Committee of School of Basic Medical Sciences, Fudan University. All husbandry and experimental procedures were reviewed and approved by the same committee (Permit Number: 20210302-137). All applicable institutional and/or national guidelines for the care and use of animals were followed. The following transgenic mouse lines were used in this study: *Nkx2.1^Cre^* (Jax 008661) (Xu et al., 2008), *Gsh2^Cre^* (Jax 025806) (Kessaris et al., 2006), *Emx1^Cre^* (Jax 005628) (Gorski et al., 2002), Ai65 (Jax 021875) (Madisen et al., 2015), and RC::FLTG (Jax 026932) (Plummer et al., 2015). The tTA2-dependent tdTomato reporter (TRE-RFP) was derived from Ai62 (Jax 022731) (Madisen et al., 2015), by removing LoxP-STOP-LoxP with *E2a-Cre* (Jax 003724). The Flp-dependent H2B-GFP reporter (HG-FRT) was derived from HG-dual (Jax 028581) via removal of loxP flanking STOP cassette by *CMV-Cre* (He et al., 2016). The *Opalin^P2A-Flpo-T2A-tTA2^* allele was generated by targeted insertion of the T2A-Flpo-P2A-tTA2 sequence immediately before the STOP codon of the endogenous *Opalin* gene using homologous recombination. Gene targeting vector was generated using PCR-based cloning approach as described before (He et al., 2016). More specifically, a 4.7-kb 5′ homology arm, a loxP flanking Neo-positive selection cassette, a T2A-Flpo-P2A-tTA2 cassette and a 2.7-kb 3′ homology arm were cloned into a building vector containing the DTA-negative selection cassette to generate the targeting vector. Targeting vector was linearized and transfected into a C57/black6 ES cell line. ES clones that survived through negative and positive selections were first screened by genomic PCR, then confirmed by Southern blotting using appropriate DIG-dUTP-labeled probes. One positive ES cell clone was used for blastocyst injection to obtain male chimera mice carrying the modified allele following standard procedures. Chimera males were bred with C57BL/6J females to confirm germline transmission by genomic PCR. The Neo selection cassette was self-excised during spermatogenesis of F0 chimeras. Heterozygous F1 siblings were bred with one another to establish the colony. Targeting vector construction, ES cell transfections and screening, blastocyst injections, and chimera breeding were performed by Cyagen.

### Genomic PCR

Genomic DNA was prepared from mouse tails. Tissue was lysed by incubation in tail lysis buffer (Viagen, 102-T) with 0.1 mg/ml proteinase K (Diamond, A100706) overnight at 55°C followed by 45 min at 90°C in an air bath to inactivate proteinase K. The lysate was cleared by centrifugation at maximum speed (21,130 G) for 15 min in a table-top centrifuge. Supernatant containing genomic DNA was used as the PCR template for amplifying DNA products. The following primers were used:

*Opalin-F*: 5′-GGCCTATGTTTGATTTCCAGCACTG-3′
*Opalin-R*: 5′-AGCACTTATGACTGCTGAGCCGTTC-3′

### Immunohistochemistry and microscopy

Mice were anesthetized by intraperitoneal injection of 1.5% sodium pentobarbital (0.09 mg/g body weight) and then intracardially perfused with saline followed by 4% paraformaldehyde in 0.1 M phosphate buffer. Following post fixation at 4°C for 24 hr, brain samples were sectioned at 30 μm using a vibratome (Leica VT1000S), or transferred into 30% sucrose in 0.1 M PB for cryoprotection, embedded in optimal cutting temperature (OCT) compound, and sectioned using a cryostat (Leica CM1950). For CC1 immunostaining, antigen retrieval was performed prior to blocking by boiling for 3 min in 10 mM citrate buffer (pH 6.0). Sections were blocked in phosphate buffered saline (PBS) containing 0.05% Triton and 5% normal donkey serum and then incubated with the following primary antibodies in the blocking solution at 4°C overnight: RFP (goat polyclonal antibody, 1:2000, SICGEN AB0081-200; rabbit polyclonal antibody, 1:2000, Rockland 600-401-379), GFP (chicken polyclonal antibody, 1:1000, Aves Labs, GFP-1020), MBP (rat polyclonal antibody, 1:500, AbD Serotec, MCA409S), CC1 (rabbit polyclonal antibody, 1:500, Oasis Biofarm, OB-PRB070, mouse polyclonal antibody, 1:300, Millipore, OP80), ASPA (rat polyclonal antibody, 1:200, Oasis Biofarm, OB-PRT005), Sox9 (rabbit polyclonal antibody, 1:2000, Chemicon, AB5535), and NeuN (mouse monoclonal antibody, 1:500, Millipore, MAB377). Sections were then incubated with appropriate Alexa fluor dye-conjugated IgG secondary antibodies (1:500, Thermo Fisher Scientific) or CF dye-conjugated IgG secondary antibodies (1:250, Sigma) in blocking solution and mounted in Aqua-mount (Southern Biotech, 0100-01). Sections were counterstained with DAPI (4',6-diamidino-2-phenylindole). Sections were imaged with confocal microscopy (Olympus FV3000), fluorescence microscopy (Nikon Eclipse Ni; Olympus VS120; Olympus VS200), and fluorescent stereoscope (Nikon SMZ25). All quantifications were performed in 2-month-old adult mice from coronal sections between Bregma +1.94 and −2.80 mm. Anatomical regions were identified according to the *Paxinos* ‘*The Mouse Brain*’ *Atlas* and the *Allen Reference Atlas*, and their areas were measured in ImageJ for density calculations, whenever applicable. For cortical regions, every fourth section within the range of selection was analyzed. For whiter matter tracts, three consecutive sections at Bregma 0.14 were analyzed. At least three brains were analyzed for each genotype. To quantify density and co-localization, cells were identified and counted in Adobe Photoshop or ImageJ in conjugation with QuPath.

### Statistical analysis

GraphPad Prism version 8.0.1 was used for statistical calculations. No statistical methods were used to predetermine sample sizes, but our sample sizes are similar to those reported in previous publications. Data collection and analysis were performed blind to the conditions of the experiments whenever possible. No animals or data points were excluded from the analysis. Normalcy was assessed using Shapiro–Wilk test. Equal variances were assessed using *F* test or Bartlett’s test. Statistical significance was tested using two-tailed unpaired *t*-test, Welch’s *t*-test, one-way analysis of variance (ANOVA), and two-way ANOVA followed by Tukey’s or Bonferroni post hoc test, wherever appropriate. Data are presented as mean ± standard error of the mean. p < 0.05 was considered significant. Significance is marked as *p < 0.05, **p < 0.01, and ***p <0.001.

## Data Availability

All data generated or analyzed during this study are included in the manuscript. Source data have been provided for Figures 1 and 2.

## Additional files

MDAR checklist

## References

[bib1] Gong L, Liu X, Wu J, He M (2022). Emerging strategies for the genetic dissection of gene functions, cell types, and neural circuits in the mammalian brain. Molecular Psychiatry.

[bib2] Gorski JA, Talley T, Qiu M, Puelles L, Rubenstein JLR, Jones KR (2002). Cortical excitatory neurons and glia, but not GABAergic neurons, are produced in the Emx1-expressing lineage. The Journal of Neuroscience.

[bib3] He M, Tucciarone J, Lee S, Nigro MJ, Kim Y, Levine JM, Kelly SM, Krugikov I, Wu P, Chen Y, Gong L, Hou Y, Osten P, Rudy B, Huang ZJ (2016). Strategies and tools for combinatorial targeting of GABAergic neurons in mouse cerebral cortex. Neuron.

[bib4] Huang H, He W, Tang T, Qiu M (2023). Immunological markers for central nervous system glia. Neuroscience Bulletin.

[bib5] Jiang W, Yang W, Yang W, Zhang J, Pang D, Gan L, Luo L, Fan Y, Liu Y, Chen M (2013). Identification of Tmem10 as a novel late-stage oligodendrocytes marker for detecting hypomyelination. International Journal of Biological Sciences.

[bib6] Kessaris N, Fogarty M, Iannarelli P, Grist M, Wegner M, Richardson WD (2006). Competing waves of oligodendrocytes in the forebrain and postnatal elimination of an embryonic lineage. Nature Neuroscience.

[bib7] Kippert A, Trajkovic K, Fitzner D, Opitz L, Simons M (2008). Identification of Tmem10/Opalin as a novel marker for oligodendrocytes using gene expression profiling. BMC Neuroscience.

[bib8] Li J, Yang F, Tian Y, Wang Z, Qi D, Yang Z, Song J, Ding J, Wang X, Zhang Z (2023). The lateral ganglionic eminence does not generate cortical oligodendrocytes. bioRxiv.

[bib9] Liu R, Jia Y, Guo P, Jiang W, Bai R, Liu C (2021). In vivo clonal analysis reveals development heterogeneity of oligodendrocyte precursor cells derived from distinct germinal zones. Advanced Science.

[bib10] Madisen L, Garner AR, Shimaoka D, Chuong AS, Klapoetke NC, Li L, van der Bourg A, Niino Y, Egolf L, Monetti C, Gu H, Mills M, Cheng A, Tasic B, Nguyen TN, Sunkin SM, Benucci A, Nagy A, Miyawaki A, Helmchen F, Empson RM, Knöpfel T, Boyden ES, Reid RC, Carandini M, Zeng H (2015). Transgenic mice for intersectional targeting of neural sensors and effectors with high specificity and performance. Neuron.

[bib11] Marques S, Zeisel A, Codeluppi S, van Bruggen D, Mendanha Falcão A, Xiao L, Li H, Häring M, Hochgerner H, Romanov RA, Gyllborg D, Muñoz Manchado A, La Manno G, Lönnerberg P, Floriddia EM, Rezayee F, Ernfors P, Arenas E, Hjerling-Leffler J, Harkany T, Richardson WD, Linnarsson S, Castelo-Branco G (2016). Oligodendrocyte heterogeneity in the mouse juvenile and adult central nervous system. Science.

[bib12] Nakahira E, Kagawa T, Shimizu T, Goulding MD, Ikenaka K (2006). Direct evidence that ventral forebrain cells migrate to the cortex and contribute to the generation of cortical myelinating oligodendrocytes. Developmental Biology.

[bib13] Naruse M, Ishino Y, Kumar A, Ono K, Takebayashi H, Yamaguchi M, Ishizaki Y, Ikenaka K, Hitoshi S (2016). The dorsoventral boundary of the germinal zone is a specialized niche for the generation of cortical oligodendrocytes during a restricted temporal window. Cerebral Cortex.

[bib14] Orduz D, Benamer N, Ortolani D, Coppola E, Vigier L, Pierani A, Angulo MC (2019). Developmental cell death regulates lineage-related interneuron-oligodendroglia functional clusters and oligodendrocyte homeostasis. Nature Communications.

[bib15] Plummer NW, Evsyukova IY, Robertson SD, de Marchena J, Tucker CJ, Jensen P (2015). Expanding the power of recombinase-based labeling to uncover cellular diversity. Development.

[bib16] Shen Z, Lin Y, Yang J, Jörg DJ, Peng Y, Zhang X, Xu Y, Hernandez L, Ma J, Simons BD, Shi S-H (2021). Distinct progenitor behavior underlying neocortical gliogenesis related to tumorigenesis. Cell Reports.

[bib17] Tripathi RB, Clarke LE, Burzomato V, Kessaris N, Anderson PN, Attwell D, Richardson WD (2011). Dorsally and ventrally derived oligodendrocytes have similar electrical properties but myelinate preferred tracts. The Journal of Neuroscience.

[bib18] Tsoa RW, Coskun V, Ho CK, de Vellis J, Sun YE (2014). Spatiotemporally different origins of NG2 progenitors produce cortical interneurons versus glia in the mammalian forebrain. PNAS.

[bib19] Winkler CC, Yabut OR, Fregoso SP, Gomez HG, Dwyer BE, Pleasure SJ, Franco SJ (2018). The dorsal wave of neocortical oligodendrogenesis begins embryonically and requires multiple sources of sonic hedgehog. The Journal of Neuroscience.

[bib20] Xu Q, Tam M, Anderson SA (2008). Fate mapping Nkx2.1-lineage cells in the mouse telencephalon. The Journal of Comparative Neurology.

[bib21] Yoshikawa F, Sato Y, Tohyama K, Akagi T, Hashikawa T, Nagakura-Takagi Y, Sekine Y, Morita N, Baba H, Suzuki Y, Sugano S, Sato A, Furuichi T (2008). Opalin, a transmembrane sialylglycoprotein located in the central nervous system myelin paranodal loop membrane. The Journal of Biological Chemistry.

[bib22] Zhang Y, Liu G, Guo T, Liang XG, Du H, Yang L, Bhaduri A, Li X, Xu Z, Zhang Z, Li Z, He M, Tsyporin J, Kriegstein AR, Rubenstein JL, Yang Z, Chen B (2020). Cortical neural stem cell lineage progression is regulated by extrinsic signaling molecule sonic hedgehog. Cell Reports.

