# Supplementary material for: Embryonic origins of forebrain oligodendrocytes revisited by combinatorial genetic fate mapping
Source: eLife. 2024 Sep 11;13:RP95406. doi: 10.7554/eLife.95406 (PMC11390105; doi:10.7554/eLife.95406)
Supplement: Figure 1—source data 1. [file elife-95406-fig1-data1.zip › Figure1-Source Data1/Figure1-Source Data1 Raw unedited blot for Figure1B.pdf]

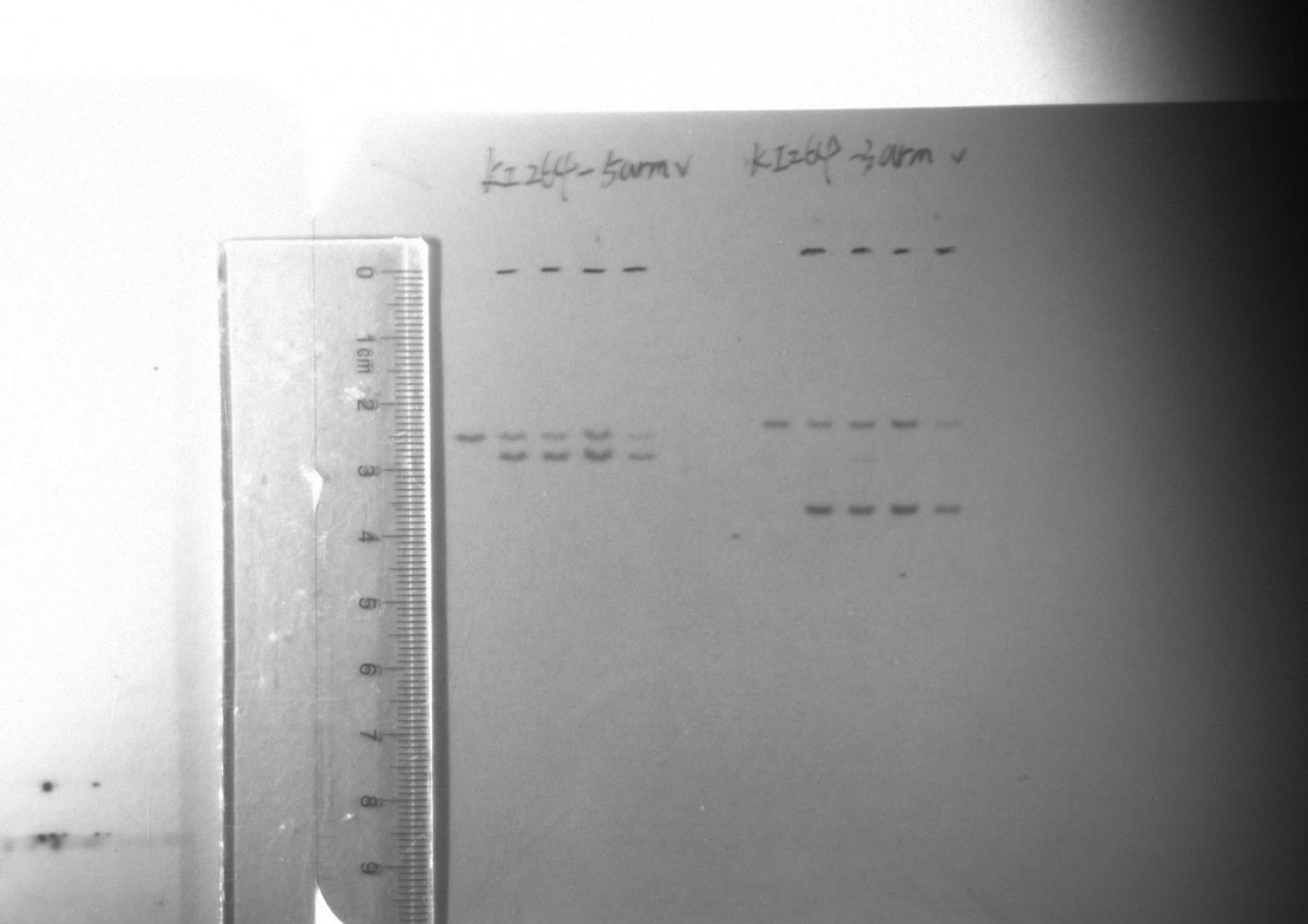
KI264-5arm v
KI264-3arm v
1cm
