# Supplementary figures and images for: Embryonic origins of forebrain oligodendrocytes revisited by combinatorial genetic fate mapping
Source: eLife. 2024 Sep 11;13:RP95406. doi: 10.7554/eLife.95406 (PMC11390105; doi:10.7554/eLife.95406)

0  
1 cm  
2  
3  
4  
5  
6  
7  
8

**BamHI**

**Opalin** *WT/WT* *Tg/WT*

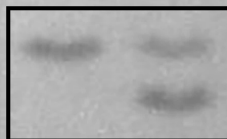

**NdeI**

**Opalin** *WT/WT* *Tg/WT*

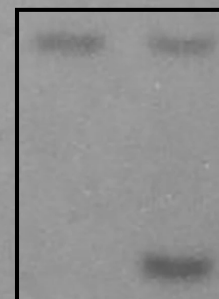

Supplement: Figure 1—source data 2. [file elife-95406-fig1-data2.zip › Figure1-Source Data2/Figure1-Source Data1 Uncropped and labeled blot for Figure1B.pdf]

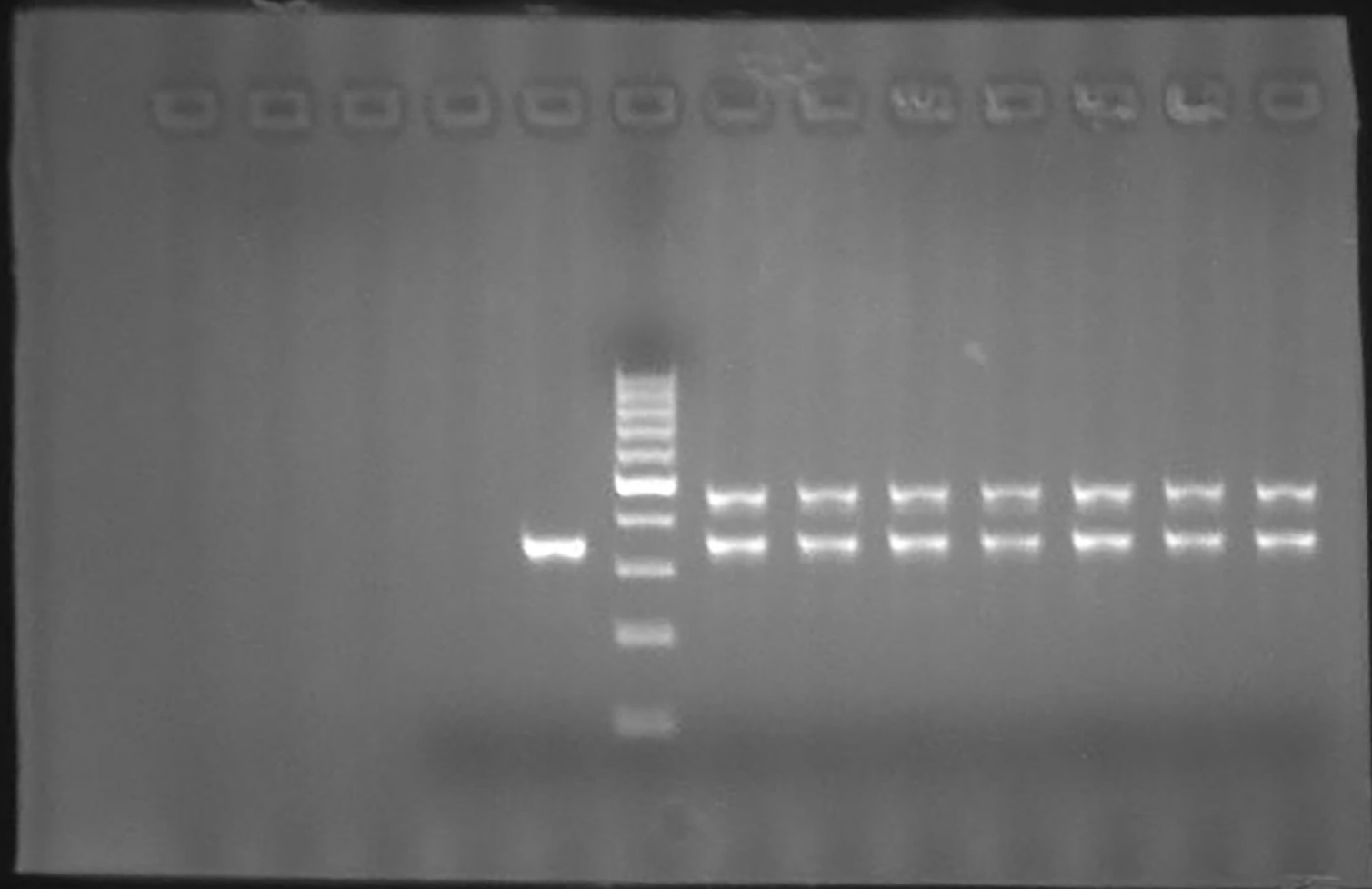

Supplement: Figure 1—source data 3. [file elife-95406-fig1-data3.zip › Figure1-Source Data3/Figure1-Source Data1 Raw unedited gel for Figure1C.pdf]

**Opalin** *WT/WT* *Flp/WT*

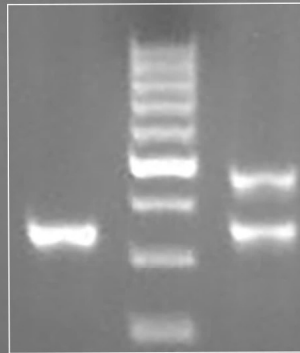

Supplement: Figure 1—source data 4. [file elife-95406-fig1-data4.zip › Figure1-Source Data4/Figure1-Source Data1 Uncropped and labeled gel for Figure1C.pdf]
